# Clinical Characterisation and Comorbidities of Acquired Generalised Lipodystrophy: A 14-Year Follow-Up Study

**DOI:** 10.3390/jcm12237344

**Published:** 2023-11-27

**Authors:** Antia Fernandez-Pombo, Teresa Prado-Moraña, Everardo Josue Diaz-Lopez, Sofia Sanchez-Iglesias, Ana I. Castro, Silvia Cobelo-Gomez, David Araujo-Vilar

**Affiliations:** 1 UETeM-Molecular Pathology Group, Department of Psychiatry, Radiology, Public Health, Nursing and Medicine, IDIS-CIMUS, University of Santiago de Compostela, 15706 Santiago de Compostela, Spain; antiafpombo@gmail.com (A.F.-P.); teresa.prado.morana@sergas.es (T.P.-M.); everardodiaz2@gmail.com (E.J.D.-L.); sofia.sanchez@usc.es (S.S.-I.); silviacobelog@gmail.com (S.C.-G.); 2 Division of Endocrinology and Nutrition, University Clinical Hospital of Santiago de Compostela, 15706 Santiago de Compostela, Spain; anaisabel0121@gmail.com; 3 CIBER Fisiopatologia de la Obesidad y la Nutricion (CIBERobn), 28029 Madrid, Spain

**Keywords:** acquired generalised lipodystrophy, adipose tissue, body composition, autoimmunity, diabetes

## Abstract

Acquired generalised lipodystrophy (AGL) is a rare disorder characterised by the gradual loss of fat that tends to generalise over time, the origin of which is still not fully clarified. The aim of this study was to offer a detailed description of seven patients with AGL (five women, 33.8 ± 18.6 years of age), evaluated over the last 14 years, in order to augment the knowledge of this disorder. The onset of the phenotype occurred during childhood and adolescence in five cases, and in adulthood in two cases. Three patients reported infections or vaccine administration prior to the development of lipodystrophy, and two subjects reported nodular swelling. The most frequent physical features were phlebomegaly, umbilical protrusion/hernia, and acanthosis nigricans. Skinfolds and body composition analysis showed the generalised absence of fat, with the exception of one patient in whom fat loss was spared in the trunk. The loss of fat in the palms/soles was observed in five subjects. Regarding metabolic comorbidities, throughout follow-up, two patients developed type 1 diabetes and one type 2 diabetes; three also presented hypertriglyceridaemia, one of whom developed acute pancreatitis, and no macrovascular complications were observed. Only one patient showed decreased complement C4. Autoimmunity was present in all cases, and six patients manifested Hashimoto’s thyroiditis, type 1 diabetes, autoimmune hepatitis, and/or celiac disease. Thus, there are certain clinical characteristics of AGL that may be considered important diagnostic criteria to differentiate this disorder from other lipodystrophy subtypes.

## 1. Introduction

Lipodystrophy syndromes are a heterogeneous group of rare diseases characterised by the selective loss of adipose tissue. They may be generalised if the loss of adipose tissue affects the whole body, or partial if only part of the body is affected. In addition, depending on their aetiology, they can also be genetic or acquired [1].

Although, according to literature and database searches, the estimated prevalence of generalised lipodystrophy was determined to be 0.2–1.0 cases per million inhabitants [2], acquired lipodystrophies are expected to be even less frequent than the corresponding genetic forms [3] and, therefore, are poorly understood syndromes.

Acquired generalised lipodystrophy (AGL), also known as Lawrence syndrome, is characterised by the gradual loss of adipose tissue, which tends to generalise over the course of weeks or even years, also affecting the palms and soles in most cases. The onset of the phenotype occurs later than in congenital generalised lipodystrophy syndromes (usually childhood or adolescence) and affects women more often than men [4,5].

The lack of adipocytes and the reduced expandability of adipose tissue in patients with generalised lipodystrophy alter their ability to buffer excess energy intake, leading to inadequate lipid storage that impairs insulin signalling. This is thought to promote insulin resistance and cause several metabolic manifestations, such as nonketotic diabetes mellitus, hypertriglyceridaemia, pancreatitis, and hepatic steatosis [4,6]. However, little is known about the evolution of comorbidities and organ abnormalities in AGL.

While molecular alterations can be identified in most cases of genetic forms of lipodystrophy, at the present time, the origin of AGL has still not been fully clarified. A classification has been proposed that divides this disorder into three subtypes: idiopathic (50%), associated with panniculitis (25%), and autoimmune (25%) [4]. In this sense, perilipin-1 autoantibodies have recently been related to the aetiology of certain cases [7,8,9]. Furthermore, another subtype of AGL related to immune checkpoint therapy (anti-PD-1 therapy) has also been described [10,11,12,13].

To date, there is no evidence of therapies able to reverse the progression of adipose tissue loss in AGL, and treatment is mainly focused on the improvement of metabolic abnormalities. In this sense, metreleptin, a human recombinant leptin, has been approved as an adjunct to diet for the treatment of complications of leptin deficiency in both acquired and congenital generalised lipodystrophy [1,14]. The correct identification and characterisation of the disease is of special relevance considering that the development of appropriate interventions may contribute to changing its natural course.

Thus, the aim of this study was to offer a detailed description of a case series of patients with AGL, including phenotype characterisation, body composition analysis, autoimmune evaluation, and the determination of organ abnormalities and disease progression, in order to augment the knowledge of this specific disorder.

## 2. Materials and Methods

### 2.1. Study Population and Design

A total of seven patients with a diagnosis of AGL referred to the reference management and treatment centre for lipodystrophies in Spain (UETeM) at the University Clinical Hospital of Santiago de Compostela over a period of 14 years (between 2008 and 2022) were included in the case series.

In order to make the diagnosis of AGL, generalised lipodystrophy was clinically confirmed by the same expert examiner in all cases. The presence of a pattern consistent with total or near-total fat loss and its onset (later in life than in congenital generalised lipodystrophy [CGL]), the absence of family history of lipodystrophy and consanguinity, and the presence of autoimmune features were taken into consideration to support the clinical diagnosis of AGL. Other causes associated with wasting or weight loss (such as cancer cachexia, malnutrition, malabsorption, anorexia nervosa, thyrotoxicosis, or chronic infections) were ruled out. Human immunodeficiency virus (HIV)-related lipodystrophy and other types of genetic or acquired lipodystrophies were excluded. The absence of pathogenic variants in known genes related to lipodystrophy was proven in all patients. The clinical diagnosis of AGL was also supported by the presence of progressive near-total fat loss according to body composition imaging in four cases using Dual-energy X-ray Absorptiometry (DXA) with a Lunar DPX model (GE Healthcare Lunar, Madison, WI, USA).

All patients followed a Mediterranean diet and lifestyle typical of Spain without any nutritional restrictions. A low-fat diet (<15% of total caloric value) was recommended if severe hypertriglyceridaemia was present. No patient performed a regulated exercise regimen or participated in competitive sports. The patients’ physical activity focused on brisk walking for around 30–60 min a day, with the exception of case #1, who led a sedentary life.

### 2.2. Clinical Data Collection

Clinical data were obtained from the patients’ electronic medical records and recorded for analysis from birth until loss of follow-up, death, or closure of the database.

Patients were evaluated by the same expert examiner following a standardised and homogeneous protocol. A physical examination was performed on all subjects, and phenotypic characteristics associated with lipodystrophy were reported. Regarding comorbidities, data collection focused on capturing metabolic and organ abnormalities related to insufficient storage capacity for excess nutrients or spillover to ectopic sites, based on the existing literature and clinical relevance. In addition, other global defects found in these patients, extending beyond the key organ systems, were also recorded.

Diabetes mellitus and diabetic neuropathy (both peripheral and autonomic) were defined according to the 2022 American Diabetes Association criteria [15,16]. Polycystic ovary syndrome (PCOS) was diagnosed based on the presence of at least two of the following three criteria: chronic anovulation, clinical and/or biochemical hyper-androgenism, and polycystic ovaries on ultrasound, after excluding other androgen excess or related disorders [17]. Hepatic steatosis was measured using high-resolution ultra-sound B-mode imaging with a convex transducer (frequency of 3.5–5 MHz) and after histological analysis through liver biopsy if indicated.

### 2.3. Anthropometry and Body Composition Analysis

Height and weight were verified after 12 h of overnight fasting with digital scales, and a stadiometer and body mass index (BMI) were calculated. Waist and hip circumferences were determined using a soft tape measure. Waist circumference was calculated, taking as a reference the superior border of the iliac crest, and hip circumference, taking as a reference the greater trochanter. The patients’ skinfolds were measured using a Lange skinfold calliper (Cambridge Scientific Industries; Cambridge, MD, USA) in the same hemibody. The mean of three consecutive determinations was obtained.

Determination of fat mass and fat-free mass (FFM), both total and segmental, was performed in a subset of patients using Dual-energy X-ray Absorptiometry (DXA) with a Lunar DPX model (GE Healthcare Lunar; Madison, WI, USA) between 8:30–10:30 a.m. after 12 h of overnight fasting, avoiding previous excessive physical effort.

### 2.4. Analytical Measurements

Laboratory results associated with metabolic complications and autoimmune disorders were documented at the end of follow-up for each patient with diagnosed AGL. Blood samples were taken between 8:00–9:00 a.m. after 12 h overnight fasting.

Fasting glucose, insulin, glycated haemoglobin (HbA1c), creatinine, transaminases, triglycerides, total and fractionated cholesterol levels, and autoantibodies were measured by standardised methods with appropriate quality control and quality assurance procedures. HOMA-IR was calculated using the following formula: (fasting glucose [mg/dL] × fasting insulin [mIU/L])/405. Serum leptin levels were determined by enzyme-linked immunosorbent assay (ELISA) (DRG International Inc.; Springfield, NJ, USA) (inter-assay coefficient of variability [CV] 3.7–9.1%, intra-assay CV 7.8–8.6%). Complement C3 and C4 were determined via immunoturbidimetry using the ADVIA analyser (Siemens, Bayer Diagnostics; Tarrytown, NY, USA).

Regarding autoantibodies, anti-glutamic acid decarboxylase (catalogue number [CN] 36010), anti-islet cell (CN EIA-4088), and anti-insulin antibodies (CN 5430) were determined by ELISA (DRG International Inc.; Springfield, NJ, USA) (inter-assay CV 3.5–6.6%, intra-assay 3.5–7.3%). The detection of anti-gastric parietal cell antibodies (CN 0832) was performed by indirect immunofluorescence incubating the serum of the patients at a 1/40 dilution (Scimedx Corporation; Denville, NJ, USA). The detection of anti-transglutaminase IgA antibodies (CN 14-55-17-01) was carried out by Fluoroenzyme immunoassay EliA Celikey IgA in a Phadia 250 instrument (Thermo Fisher Scientific; Waltham, MA, USA), following the manufacturer’s instructions. Anti-thyroid peroxidase (CN 10995466) and anti-thyroglobulin antibodies (CN L2KTG2) were determined by chemiluminescence using an ADVIA Centaur assay and an IMMULITE 2000 analyser, respectively (Siemens, Bayer Diagnostics; Tarrytown, NY, USA). Anti-adrenal (CN 5475) and anti-nuclear (CN 26103) antibodies were analysed in an external laboratory (Reference Laboratory S.A.) using immunofluorescence assays.

### 2.5. Molecular Analysis

The search for variants in 26 genes involved in the aetiology of congenital lipodystrophies was conducted in all the patients evaluated by next-generation sequencing (NGS) (Ion Torrent System, Thermo Fisher Scientific; Waltham, MA, USA) of the entire coding region of the genes and the flanking intronic regions (*ADRA2A*, *AGPAT2*, *AKT2*, *BANF1*, *BLM*, *BSCL2*, *CAV1*, *CIDEC*, *ERCC6*, *ERCC8*, *FBN1*, *KCNJ6*, *LIPE*, *LMNA*, *MFN2*, *PCYT1A*, *PIK3R1*, *PLIN1*, *POLD1*, *POLR3A*, *PPARG*, *PSMB8*, *PTRF*, *SPRTN*, *WRN*, *ZMPSTE24*). No pathogenic variants in these genes have been detected in any of the cases with AGL.

### 2.6. Statistical Analysis

Descriptive analysis was undertaken.

## 3. Results

Seven patients with AGL (five women, 33.8 ± 18.6 years of age) were included in the series. Although no patient had a family history of lipodystrophy, consanguinity was observed in two cases.

**Figure 1:**
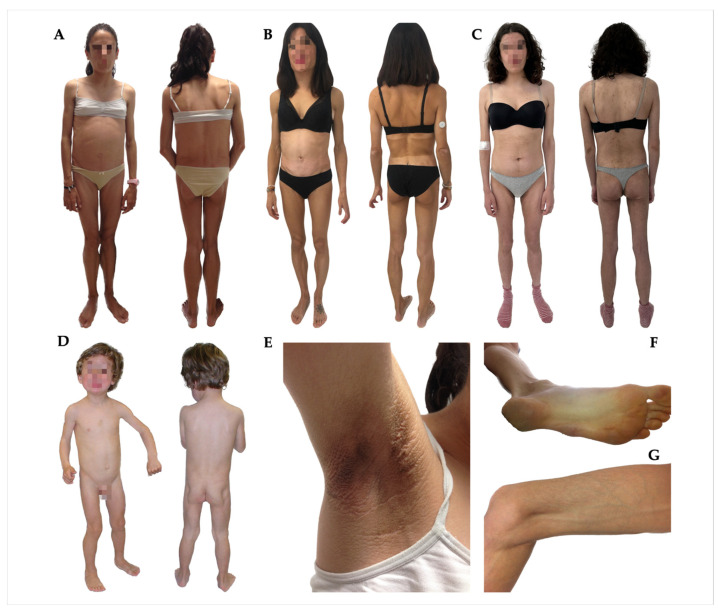
Clinical features of patients with acquired generalised lipodystrophy. Near-total generalised loss of adipose tissue observed in patient #2 at 13 years of age (**A**), patient #5 at 39 years of age (**B**), patient #7 at 18 years of age (**C**), and patient #6 at 2 years and 10 months of age (**D**). Patient #6 preserves facial fat. Acanthosis nigricans in patient #2 (**E**). Foot of patient #5 showing severe loss of subcutaneous fat (**F**). Loss of fat in the lower limb of patient #2 along with phlebomegaly (**G**).

The onset of the phenotype was evident during childhood in four cases (patients #1, #2, #5, and #6), in adolescence in one case (patient #7), and in adulthood in two cases (patients #3 and #4) (Table 1). Patient #2 reported infections prior to the development of generalised lipodystrophy, specifically recurrent otitis and parotitis. Patient #5 referred to the appearance of the phenotype after having chickenpox, and patient #6 after the administration of the varicella vaccine. In addition, two patients (patients #1 and #4) reported a previous history of nodular swelling, which resolved spontaneously. The most frequent physical feature was phlebomegaly (six out of seven patients), followed by umbilical protrusion/hernia and signs of insulin resistance such as acanthosis nigricans (in four patients) (Figure 1). No acromegaloid features were observed. Other phenotypic characteristics of these subjects are also shown in Table 1.

**Figure 2:**
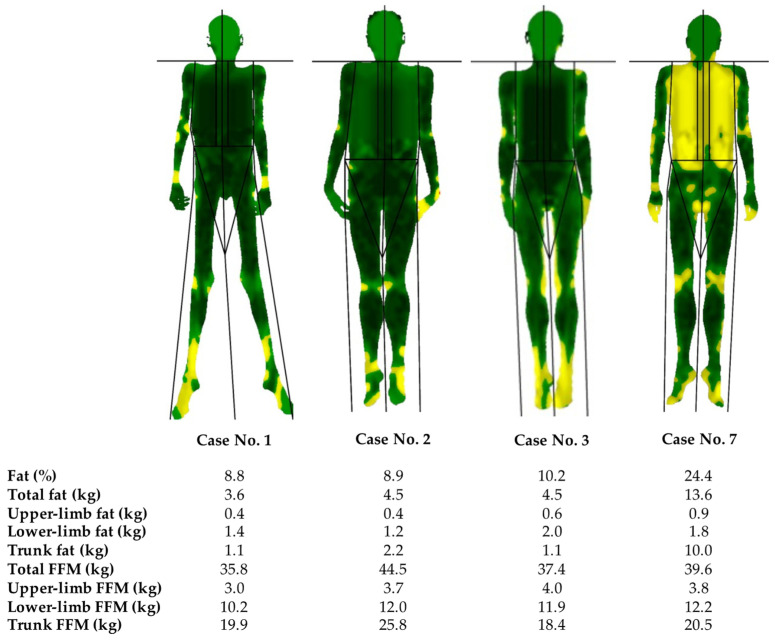
Body composition analysis determined by Dual-energy X-ray Absorptiometry of subjects with acquired generalised lipodystrophy. Green represents an area of low-level % fat (0–25%), yellow an area of medium-level % fat (25–60%), and red an area of high-level % fat (60–100%). Near-total generalised loss of adipose tissue can be observed in patients #1–3, with less-pronounced fat loss in the trunk in patient #7. FFM: fat-free mass.

Both the skinfolds and the body composition analysis via DXA showed the characteristic generalised absence of adipose tissue in these patients, with no notable alterations in FFM distribution in the overall sample. The percentage of body fat was 8.8–10.2%. The exceptions were patients #4 and #7, in whom fat loss was found to be spared in the limbs and truncal area, respectively. In this sense, patient #4 progressively lost subcutaneous fat from the rest of the body during follow-up. Regarding patient #7 (presenting with a total fat of 24.4%), taking into account her relative recent onset of the phenotype, fat loss of adipose tissue was also considered to be still in the process of progressively becoming generalised, especially when comparing with pictures of when she was 15 years of age. These results can be observed in more detail in Figure 2 and Table 2. In addition, the loss of adipose tissue in the palms and/or soles was observed in five out of the seven subjects, and facial fat was preserved in three cases (Figure 1).

As for the main comorbidities (Table 1), three patients developed diabetes mellitus, and two developed prediabetes. Regarding microvascular complications, there were two cases of albuminuria and one case of diabetic peripheral neuropathy. No macrovascular complications were observed in this cohort. Hepatomegaly and/or splenomegaly were reported in three subjects, one of whom (patient #2) also showed moderate elevation of transaminases throughout follow-up. Ultrasound-guided liver biopsy in this patient showed lymphoplasmacytic infiltrates in portal spaces and interface hepatitis with lobular inflammation, balonisation, fibrosis with septa and bridges, and steatosis with a diffuse macrovacuolar predominance of around 60%. She was subsequently diagnosed with autoimmune hepatitis. An episode of acute pancreatitis was also reported in one subject. Two out of the five women presented with amenorrhea, with one of them being diagnosed with PCOS. In addition, patient #1 also developed mesangial proliferative glomerulonephritis and mucociliary dysfunction. He died at the age of 25 due to a respiratory tract infection. Regarding autoimmune disorders, apart from patient #2 being diagnosed with autoimmune hepatitis, patients #1 and #5 were also diagnosed with type 1 diabetes mellitus, patients #3 and #6 developed celiac disease, and patients #4 and #6 had a history of Hashimoto’s thyroiditis with overt hypothyroidism.

Autoimmunity analysis is shown in Table 3. Autoimmunity was present in all reported cases. In addition, in four subjects, more than one type of autoantibody was detected. Both beta-cell autoimmunity and autoimmune thyroid disease were present in two cases. Other autoantibodies detected in separate patients were anti-nuclear, anti-transglutaminase, anti-parietal cell, and anti-adrenal antibodies. Rheumatoid factor was found to be positive in one case. Patients #3 and #6 also showed positive HLA DQA1*0501 and positive HLA DQB1*0201. Other autoantibodies measured in patients #4 and #6 were anti-mitochondrial, anti-smooth muscle, anti-extractable nuclear antigen, anti-native DNA, anti-cyclic citrullinated peptides, anti-cardiolipin, and anti-beta-2-glycoprotein antibodies, with a negative result.

Other laboratory values are shown in Table 4, which correspond to the analytical results at the last visit of the patients to the centre at the end of follow-up. Patients with diagnosed diabetes mellitus (#1, #2, and #5) presented poor metabolic control at this last visit, even with multiple-dose insulin therapy in two of the cases, with mean HbA1c levels of 8.6 ± 1.6%. Triglyceride and transaminase levels were variable, with three patients presenting hypertriglyceridaemia without remarkable alterations in the rest of the lipid profile, with the exception of low high-density lipoprotein cholesterol levels in two cases. Thus, due to poor metabolic control in patients #1 and #2 (taking into account both HbA1c and triglyceride levels shown in Table 4), the decision was subsequently taken to initiate treatment with recombinant human leptin. As expected, serum leptin levels were diminished in accordance with the fat loss. On the other hand, all subjects showed vitamin D deficiency accompanied by normal calcium levels, with two of the cases already receiving vitamin D3 supplementation at the time of analysis. Only patient #2 showed decreased complement C4 levels (7.7 mg/dL [normal values: 10–40 mg/dL]), whereas for the rest of the patients in whom complement C3 and complement C4 levels were evaluated, these were found to be within the normal range.

Analytical parameters without (patients #3–7) or prior to (patients #1 and #2) treatment with recombinant human leptin at the last visit to the centre of the subjects with diagnosed acquired generalised lipodystrophy at the end of follow-up. HbA1c: glycated haemoglobin; LDL-C: low-density lipoprotein cholesterol; HDL-C: high-density lipoprotein cholesterol; AST: aspartate aminotransferase; ALT: alanine aminotransferase; GGT: γ-glutamyl transpeptidase; PTH: parathormone; 25(OH)D: 25-hydroxy vitamin D.

## 4. Discussion

In the current case series, the clinical picture, the pattern of body fat loss, and the organ abnormalities of seven patients with a diagnosis of AGL evaluated over the last 14 years are described in order to contribute to augmenting the knowledge of this ultra-rare disorder.

AGL is characterised by the generalised loss of adipose tissue, as shown in the body composition analysis via DXA and skinfold measurement in this series. The percentage of body fat was 8.8–10.2% and, therefore, consistent with that previously reported in the literature (0.3–14.7%) [4,18,19]. However, marked heterogeneity has been described, with fat loss occurring only in the limbs and face but not in the trunk and intra-abdominal regions in a few cases [4], and generalising over weeks or even years, as is the case of two of the patients reported here. The loss of adipose tissue from the palms and soles in these subjects can also be considered a supportive criterion for the diagnosis of AGL, previously identified in about one-third and one half of the patients, respectively [4], and in a slightly higher proportion in the current cohort. In addition, in contrast to subjects with CGL [20], individuals with AGL have shown well-preserved bone marrow fat in previous studies [4,19,21]. Another differential feature is the age at the onset of fat loss, which generally manifests during childhood and adolescence [22,23,24], although it can also occur in adulthood and, therefore, later than in CGL syndromes [25]. Women are likewise considered to be affected more often, in a 3:1 ratio in comparison with men [5].

To date, the specific origin of this syndrome is still unknown. An aetiology classification was proposed several years ago, dividing AGL into three main subtypes: autoimmune (25%), panniculitis-associated (25%), and idiopathic (50%) [4]. In fact, in relation to the autoimmune origin of the disease, the first anti-adipocyte autoantibody predominantly directed against the lipid droplet protein perilipin 1 has recently been identified in these patients [7,8,9]. In addition, another subtype related to immune checkpoint therapy (anti-PD-1 therapy) has also recently been described [10,11,12,13]. In our cohort, autoantibody positivity was reported in all patients, with only one case reporting no clinical evidence of related autoimmune disorders and with four patients presenting more than one type (positivity for anti-glutamic acid decarboxylase, anti-islet cell, anti-insulin, anti-thyroid peroxidase, anti-nuclear, anti-transglutaminase, anti-parietal cell, and anti-adrenal antibodies, as well as rheumatoid factor, was observed). These data could suggest a higher prevalence of the subtype associated with autoimmunity than previously reported. On the other hand, as expected, leptin levels were drastically diminished in these individuals. Low leptin levels have been associated with the activation of the classical complement pathway and low levels of complement C4 in some cases of AGL throughout the literature [26,27] and in one patient in this case series. This inappropriate complement activation may also contribute to the development of autoimmune disorders. In addition, it is already known that, in organ-specific autoimmune diseases, infections can act as a trigger [28]. In fact, recurrent otitis and parotitis were reported prior to the development of generalised lipodystrophy in one case, and two patients related the appearance of the phenotype after having chickenpox or the varicella vaccine. As for the clinical translation of autoimmunity, in the current cohort, six patients with AGL manifested Hashimoto’s thyroiditis with overt hypothyroidism, type 1 diabetes, autoimmune hepatitis, and/or celiac disease. Although it has been stated that juvenile dermatomyositis may be particularly associated with AGL [4], no cases were reported here. Furthermore, two subjects (28%) referred to a previous history of spontaneously resolved nodular swelling preceding the onset of lipodystrophy, although it has not been possible to obtain a histological confirmation of panniculitis. Both subjects also shared autoimmune disorders. In this sense, an overlap between panniculitis and autoimmune disease as possible aetiopathogenetic mechanisms of AGL has previously been reported [4].

As far as phenotypical characteristics are concerned, the most frequently reported feature was phlebomegaly (six out of seven patients), followed by umbilical protrusion/hernia (four patients), the latter being a characteristic also specific to patients with CGL [1,29]. However, unlike these subjects, acromegaloid facial features were not observed in our AGL sample and are considered to be rare [30]. As for metabolic comorbidities, insulin resistance, nonketotic diabetes, hypertriglyceridaemia, and consequent pancreatitis are common [4,6]. In our cohort, insulin resistance stigmata was present in three patients; three subjects manifested diabetes with poor metabolic control, and two had prediabetes. Two subjects with diagnosed diabetes and one patient with prediabetes had positive autoimmunity. Thus, in these individuals, early-onset diabetes may appear both as a consequence of extreme fat loss and as a result of the destruction of pancreatic beta cells related to the autoimmune process. On the other hand, only a few patients with AGL have been reported to develop coronary disease in the literature [31]. In fact, while microvascular complications such as albuminuria and diabetic peripheral neuropathy were present in two of our patients, no macrovascular complications were observed in the overall sample.

## 5. Conclusions

There are several clinical features that make it possible to differentiate AGL from other lipodystrophy subtypes, and, although to date the origin of AGL has not been fully clarified, a higher frequency of associated autoimmunity is reported. Taking into account the rarity of this specific disorder, international cooperation through large registries is needed in order to present a more objective picture of the disease and its pathogenic mechanisms.

## Figures and Tables

**Table 1:** Demographic data, phenotypic features, and comorbidities of patients with acquired generalised lipodystrophy.

Case No.	Age (Years)	Gender	Onset of Phenotype	Consanguinity	Altered Fat Distribution	Clinical Features	Comorbidities
1	25	M	Childhood (8 years)	Yes	Generalised loss of fat. Palms and soles fat loss; facial fat loss	Acanthosis nigricans, prognathism, phlebomegaly, umbilical hernia, polyphagia	Type 1 diabetes, albuminuria, diabetic neuropathy, hypertriglyceridaemia, hypertension, hepatomegaly, pancreatitis, hypothyroidism, mesangial proliferative glomerulonephritis, and mucociliary dysfunction
2	23	F	Childhood (6 years)	No	Generalised loss of fat. Palms fat loss, soles, and facial fat preserved	Acanthosis nigricans, phlebomegaly, and umbilical hernia. polyphagia	Type 2 diabetes, albuminuria, hypertriglyceridaemia, hepatomegaly, splenomegaly, and autoimmune hepatitis
3	46	F	Adulthood (35 years)	No	Generalised loss of fat. Palms, soles preserved, and facial fat loss	Phlebomegaly	Amenorrhea, celiac disease
4	65	F	Adulthood (44 years)	No	Generalised loss of fat. Palms, soles, and facial fat preserved	Muscle pain	Splenomegaly, primary hypothyroidism
5	44	F	Childhood (3 years)	No	Generalised loss of fat. Palms and soles fat loss; facial fat loss	Acanthosis nigricans, phlebomegaly, and umbilical hernia	Type 1 diabetes, amenorrhea
6	16	M	Childhood (2 years)	No	Generalised loss of fat. Palms and soles fat loss; facial fat preserved	Phlebomegaly, umbilical hernia	Prediabetes, primary hypothyroidism, and celiac disease
7	19	F	Adolescence (16 years)	Yes	Generalised loss of fat. Palms and soles fat loss; facial fat loss	Acanthosis nigricans, phlebomegaly, and hirsutism	Prediabetes, amenorrhea, and PCOS

M: male; F: female; PCOS: polycystic ovary syndrome.

**Table 2:** Anthropometric measurements in patients with acquired generalised lipodystrophy.

	Case No. 1	Case No. 2	Case No. 3	Case No. 4	Case No. 5	Case No. 6	Case No. 7
Weight (kg)	40.1	51.5	43.8	46.5	47.0	50.0	56
BMI (kg/m^2^)	12.1	19.1	15.1	21.2	17.3	17.8	19.4
Waist perimeter (cm)	74	81	64	NA	NA	52	81
Hip perimeter (cm)	78	77	77	NA	NA	52	86
Triceps skinfold (mm)	2.8	3.0	4.0	14.0	4.0	4.3	4.0
Biceps skinfold (mm)	2.0	3.0	1.8	3.8	3.0	2.2	3.0
Subscapular skinfold (mm)	4.0	6.0	4.5	6.0	7.0	5.8	11.0
Suprailiac skinfold (mm)	3.8	4.0	3.6	NA	4.0	5.6	8.0
Thigh skinfold (mm)	3.2	4.0	4.2	20.0	4.0	5.2	6.0
Calf skinfold (mm)	3.2	3.5	3.0	11.0	3.0	3.9	4.5

BMI: body mass index; NA: not available.

**Table 3:** Autoimmunity analysis in patients with acquired generalised lipodystrophy.

Case No.	Anti-GAD	Anti-IA2	Anti-IAA	Anti-TPO	Anti-Tg	ANA	ATA	APCA	ADR	RF
1	+	-	+	-	-	NA	-	NA	+	NA
2	-	-	-	-	-	-	-	+	NA	NA
3	-	-	-	-	-	NA	+	-	NA	NA
4	-	-	-	+	-	+	-	-	-	NA
5	+	+	-	-	-	+	-	NA	NA	-
6	+	-	-	+	-	-	+	-	-	NA
7	-	-	-	-	-	NA	NA	NA	-	+

+: positive result; -: negative result; NA: not available; Anti-GAD: anti-glutamic acid decarboxylase; anti-IA2: anti-islet cell; anti-IAA: anti-insulin; anti-TPO: anti-thyroid peroxidase; anti-Tg: anti-thyroglobulin; ANA: anti-nuclear antibodies; ATA: anti-transglutaminase antibodies; APCA: anti-parietal cell antibodies; ADR: anti-adrenal antibody; RF: rheumatoid factor.

**Table 4:** Biochemistry at the last visit of patients with diagnosed acquired generalised lipodystrophy.

	Case No. 1	Case No. 2	Case No. 3	Case No. 4	Case No. 5	Case No. 6	Case No. 7
Fasting glucose (mg/dL) (70–100)	186	277	83	87	109	93	95
Insulin (mIU/L) (1.5–18.5)	NA	101.1	3.7	NA	13.7	9.4	144.3
HOMA-IR (<2.0)	NA	69.1	0.8	NA	3.7	2.2	33.8
HbA1c (%) (3.5–5.6)	8.1	10.4	5.4	5.7	7.3	5.8	5.4
Total cholesterol (mg/dL) (150–255)	135	215	154	180	197	141	133
LDL-C (mg/dL) (55–125)	42	92	71	56	52	35	29
HDL-C (mg/dL) (34–91)	17	24	71	56	52	35	29
Triglycerides (mg/dL) (27–150)	380	1249	58	48	68	102	256
Total bilirubin (mg/dL) (0.2–1.3)	0.5	0.3	0.6	0.3	0.4	0.4	0.3
AST (IU/L) (10–40)	23	98	21	25	23	24	23
ALT (IU/L) (3–41)	28	186	29	16	24	23	46
GGT (IU/L) (8–73)	12	147	17	15	20	18	23
Creatinine (mg/dL) (0.4–1.3)	0.6	0.3	0.7	0.6	0.6	0.9	0.6
Calcium (mg/dL) (8.5–11.0)	8.6	9.4	9.2	8.8	10.4	9.6	9.1
Phosphate (mg/dL) (2.7–5.0)	NA	4.9	NA	4.6	4.5	4.7	3.6
PTH (pg/mL) (14–72)	44	20	29	NA	14	NA	NA
25(OH)D (ng/mL) (deficiency <30)	5	24	7	12	26	32	8
Leptin (μg/L) (3.60–11.10)	0.10	0.05	NA	NA	NA	0.10	2.70
Complement C3 (mg/dL) (85–180)	NA	146.3	NA	88.0	113.0	151.0	137.0
Complement C4 (mg/dL) (11–42)	NA	7.7	NA	17.0	16.0	32.0	20.8
Treatment at the time of analysis	Insulin glargine and lispro, levothyroxine	Metformin, insulin glargine and lispro, fenofibrate, enalapril, losartan	No medication	Levothyroxine	Metformin, sitagliptin, vitamin D3	Metformin, levothyroxine, vitamin D3	Fenofibrate

## Data Availability

The data presented in this study are available on request from the corresponding author. These data are not publicly available due to privacy concerns relating to personal clinical and genetic information.
